# Epidemiology of Dermatophytosis in Tehran, Iran: A Ten-year Retrospective Study

**DOI:** 10.34172/aim.2022.82

**Published:** 2022-08-01

**Authors:** Saeed Aref, Sara Nouri, Hamideh Moravvej, Mojtaba Memariani, Hamed Memariani

**Affiliations:** ^1^Skin Research Center, Shahid Beheshti University of Medical Sciences, Tehran, Iran

**Keywords:** Dermatophyte, Epidermophyton, Iran, Microsporum, Trichophyton

## Abstract

**Background:**

Dermatophytosis still remains a major public health concern worldwide, particularly in developing countries. This study was undertaken to determine the etiological and epidemiological factors of dermatophyte infections in Tehran, Iran.

**Methods:**

A total of 1530 patients clinically suspected of cutaneous fungal infections were examined in two hospitals over a period of 10 years (2010–2020). Samples were analyzed using direct microscopic examination and culture. Data regarding age, gender, and clinical manifestations were also recorded.

**Results:**

Out of 1530 cases examined, dermatophytes were detected in 493 (32.2%) patients. Of these patients, 288 (58.4%) were males and 205 (41.6%) were females. The most affected age group was the 25–44 years old (31.6%). Tinea corporis (n=134) was the most prevalent type of ringworm, followed by tinea cruris (n=131), tinea pedis (n=90), tinea manuum (n=65), tinea unguium (n=29), tinea faciei (n=20), tinea capitis (n=18), and tinea barbae (n=2). Both tinea cruris (*P*<0.001) and tinea pedis (*P*=0.002) had a significant association with male gender. As for etiological agents, *Trichophyton mentagrophytes* (29.0%) was the most frequent isolate, followed by *Trichophyton tonsurans *(25.8%), *Trichophyton rubrum* (25.3%), *Epidermophyton floccosum* (6.9%), *Trichophyton verrucosum* (4.9%), *Microsporum audouinii* (4.5%), *Microsporum canis* (2.0%), and *Trichophyton violaceum* (1.6%).

**Conclusion:**

Dermatophytes are still the prevailing causes of fungal infection of the skin, hair, and nails in Iran. Further studies with larger samples sizes and inclusion of diverse locations would yield more accurate results.

## Introduction

 Dermatophytosis, also known as ringworm, is a disease in which keratinized tissues, such as the epidermis, hair, and nails, become infected.^1^ The disease is estimated to afflict around one quarter of the world’s population. Dermatophytosis is caused by dermatophytes, a group of closely related filamentous fungi that invade and digest keratin.^2^ Dermatophytes are amongst the most pervasive causes of skin disease across the globe, and the true prevalence is likely underestimated. These pathogens are classified in three genera, namely *Epidermophyton, Microsporum,* and *Trichophyton*.^3^ On the basis of their natural habitat and host preferences, dermatophytes are often categorized into anthropophilic, zoophilic, and geophilic species.^4^

 Dermatophytic infections are usually restricted to the non-living, cornified layers of patients. They are not typically as life-threatening as invasive mycoses, and typically affect normal individuals, but accurate distinction of causative agents is essential for proper treatment and control of the infection.^5^ Nowadays, dermatophytes are increasingly becoming more prevalent as a result of the widespread administration of immunosuppressive drugs for treating non-infectious conditions.^6^ Traditionally, dermatophytosis has been named by appending the Latin name of the site of infection after the word “tinea”. For instance, tinea capitis refers to infection of the head region or tinea pedis, also known as athlete’s foot, involves any part of the foot.^7^

 The last decades have witnessed a significant change in the distribution of dermatophytes isolated from clinical samples.^8^ For instance, *Epidermophyton floccosum*, *Microsporum audouinii*, and *Trichophyton schoenleinii* were the main pathogens of superficial fungal diseases a century ago. Nowadays, however, these species are largely replaced by *T. rubrum*, *T. interdigitale*, *T. tonsurans*, and *M. canis* in many countries.^9^ Even within the same country, the causative agent of the disease and its incidence might differ from one location to the next. In general, different factors such as geographic location, hygiene conditions, climate (temperature, humidity, wind, etc), occupation, contact with animals, socioeconomic alterations, and migration are responsible for discrepancies in the epidemiology of dermatophytosis.^10^

 Thus far, there have been only few epidemiological studies on the human dermatophytosis in Iran. In recent years, dermatophytosis has been increasingly reported as the dominant superficial mycosis in the developed world.^11^ The availability of scanty data on the prevalence and associated epidemiological factors of dermatophytosis in Iran prompted us to carry out the present survey in order to characterize the mycological and clinical aspects of dermatophytosis in Tehran, Iran.

## Material and Methods

 This study was a retrospective analysis of the data obtained from records of the Department of Dermatology of Shohada-e-Tajrish and Loghman-e-Hakim hospitals in Tehran, Iran. A total of 1530 clinical samples were retrieved from suspected patients during a 10-year period (2010–2020). Skin scrapings, scalp scales, plucked hairs, nail clippings, and subungual debris were collected to obtain material for direct microscopic examination and culture.^12^ Data on age, gender, clinical manifestations, and site of involvement were also recorded for each subject. The effect of gender and age variables on the incidence of different infections was tested by chi-square (χ^2^) test in SPSS Statistics 20.0 (SPSS Inc. Chicago, Illinois, USA). Statistical significance was achieved when *P* ˂ 0.05.

 Briefly, one portion of each clinical sample was mounted in a drop of 10% (*w*/*v*) KOH on a clean microscopic slide to verify the presence of fungal elements. A second portion was inoculated into Sabouraud’s dextrose agar medium (Merck Co. Darmstadt, Germany) supplemented with chloramphenicol and cycloheximide according to the manufacturer’s instructions. Incubation at 25–30°C was performed for at least 4 weeks to detect dermatophytes. The plates were examined twice a week for any fungal growth. Identification of dermatophyte species was mainly based on their macroscopic and microscopic characteristics. For macroscopic identification, colony morphology, color, texture, rate of growth, and pigmentation were evaluated.^13^ As for microscopic examination, lactophenol cotton blue mount of slide cultures revealed the structure and shape of the hyphae as well as the presence and arrangement of microconidia and macroconidia.^14^

## Results

 Out of 1530 clinically suspected cases examined, 493 (32.2%) patients were mycologically positive by direct microscopy and/or culture during 2010 to 2020. Of these patients, 288 (58.4%) were males and 205 (41.6%) were females. The male-to-female ratio was 1.4:1. The mean ( ± SD) age of the patients was 36.9 ( ± 17.2) years. The age range was 4 to 90 years.

 Tinea corporis (n = 134) was the most common type of cutaneous mycotic infection, followed by tinea cruris (n = 131), tinea pedis (n = 90), tinea manuum (n = 65), tinea unguium (n = 29), tinea faciei (n = 20), tinea capitis (n = 18), and tinea barbae (n = 2). In addition, tinea incognito was diagnosed in four patients. The frequencies of etiologic agents based on their locations are shown in Table 1. For instance, the *Trichophyton* species were most frequently (114/131, 87%) isolated from the groin with the main etiological agents of tinea cruris. On the other hand, *T. tonsurans* was the most common pathogen in tinea corporis (65/134, 48.5%), tinea manuum (31/65, 47.7%), and tinea capitis (5/18, 27.8%).

**Table 1:** Isolated Dermatophyte Species According to the Clinical Features in Tehran, Iran (2010–2020)

**Dermatophyte Species**	**Number of Isolates (%)**								
	**Tinea Barbae**	**Tinea Faciei**	**Tinea Cruris**	**Tinea Pedis**	**Tinea Manuum**	**Tinea Corporis**	**Tinea Unguium**	**Tinea Capitis**	**Tinea Incognito**
*Trichophyton rubrum* (n = 125)	0 (0)	0 (0)	43 (32.8)	30 (33.3)	11 (16.9)	25 (18.7)	11 (37.9)	4 (22.2)	1 (25)
*Trichophyton tonsurans* (n = 127)	0 (0)	13 (65)	4 (3.1)	3 (3.3)	31 (47.7)	65 (48.5)	5 (17.2)	5 (27.8)	1 (25)
*Trichophyton mentagrophytes* (n = 143)	2 (100)	5 (25)	63 (48.1)	31 (34.5)	10 (15.4)	20 (14.9)	9 (31.0)	2 (11.1)	1 (25)
*Trichophyton violaceum* (n = 8)	0 (0)	0 (0)	2 (1.5)	3 (3.3)	1 (1.5)	1 (0.7)	0 (0)	1 (5.5)	0 (0)
*Trichophyton verrucosum* (n = 24)	0 (0)	0 (0)	2 (1.5)	9 (10.0)	7 (10.8)	3 (2.2)	2 (6.9)	1 (5.5)	0 (0)
*Epidermophyton floccosum* (n = 34)	0 (0)	0 (0)	13 (9.9)	8 (8.9)	4 (6.1)	5 (3.7)	2 (6.9)	1 (5.5)	1 (25)
*Microsporum audouinii* (n = 22)	0 (0)	0 (0)	1 (0.8)	3 (3.3)	0 (0)	14 (10.5)	0 (0)	4 (22.2)	0 (0)
*Microsporum canis* (n = 10)	0 (0)	2 (10)	3 (2.3)	3 (3.3)	1 (1.5)	1 (0.7)	0 (0)	0 (0)	0 (0)
Total (n = 493)	2 (100)	20 (100)	131 (100)	90 (100)	65 (100)	134 (100)	29 (100)	18 (100)	4 (100)

Table 2 shows the frequencies of dermatophytosis infections with regard to gender. Gender showed a significant effect on the prevalence of some clinical presentations. Both tinea cruris (*P* < 0.001) and tinea pedis (*P*= 0.002) had significant associations with gender. These conditions were more often observed in men than women. Although tinea manuum was more prevalent in females than males, we did not find any significant association between tinea manuum and patients’ sex (*P*= 0.535).

**Table 2:** Frequency and Distributions of Clinical Manifestations by Gender (n = 493)

**Clinical Manifestation**	**Male**	**Female**	**Total, n (%)**	***P *** **Value** ^a^
Tinea barbae	2	0	2 (0.4)	0.513^b^
Tinea faciei	12	8	20 (4.1)	0.371
Tinea cruris	86	45	131 (26.6)	< 0.001
Tinea pedis	60	30	90 (18.2)	0.002
Tinea manuum	30	35	65 (13.2)	0.535
Tinea corporis	70	64	134 (27.2)	0.604
Tinea unguium	16	13	29 (5.9)	0.577
Tinea capitis	11	7	18 (3.6)	0.345
Tinea incognito	1	3	4 (0.8)	0.312^b^
Total	288 (58.4)	205 (41.6)	493 (100)	–

^a^ *P* value from Chi-square test for prevalence of infections between genders. ^b^ *P* value from Fisher’s exact test.

 Clinical manifestation in relation to age group was highest with the age group 25–44 years old (n = 156), followed by the age group 15–24 years old (n = 145), the age group 45–64 years old (n = 102), the age group 1–14 years old (n = 68), and the age group ≥ 65 years old (n = 22). As for tinea corporis (the most prevalent infection), the age group 15–24 years had the highest number (42/134, 31.3%) of cases. By contrast, tinea cruris, the second most common clinical manifestation (n = 131), was the highest (63/131, 48.1%) in patients of the age group 25–44 years. On the whole, the prevalence of the majority of the clinical forms differed significantly in patients of various age groups (*P*< 0.05). The frequencies of clinical manifestations in different age groups are presented in Table 3.

**Table 3:** Distribution of Isolated Dermatophytes on the Basis of Age Groups (Year) in Tehran, Iran (2010–2020)

**Clinical Manifestations**	**Age Groups (%)**					***P *** **Value** ^a^
	**1–14**	**15–24**	**25–44**	**45-64**	**≥65**	
Tinea barbae (n = 2)	0 (0)	0 (0)	1 (0.6)	1 (1.0)	0 (0)	-
Tinea faciei (n = 20)	6 (8.8)	9 (6.2)	3 (1.9)	2 (2.0)	0 (0)	0.014
Tinea cruris (n = 131)	4 (5.9)	43 (29.7)	63 (40.4)	18 (17.6)	3 (13.6)	< 0.001
Tinea pedis (n = 90)	8 (11.8)	13 (9.0)	35 (22.5)	33 (32.3)	1 (4.6)	< 0.001
Tinea manuum (n = 65)	8 (11.8)	28 (19.3)	17 (10.9)	12 (11.8)	0 (0)	< 0.001
Tinea corporis (n = 134)	27 (39.7)	42 (28.9)	30 (19.2)	23 (22.5)	12 (54.5)	0.001
Tinea unguium (n = 29)	3 (4.4)	6 (4.1)	5 (3.2)	12 (11.8)	3 (13.6)	0.051
Tinea capitis (n = 18)	12 (17.6)	3 (2.1)	1 (0.6)	1 (1.0)	1 (4.6)	< 0.001
Tinea incognito (n = 4)	0 (0)	1 (0.7)	1 (0.6)	0 (0)	2 (9.1)	–
Total (n = 493)	68 (100)	145 (100)	156 (100)	102 (100)	22 (100)	–

^a^ *P* value from Chi-square test between different age groups.

## Discussion

 Throughout much of the Middle East countries, dermatophytosis still remains a widespread public health issue. Correct identification of etiological agents is of paramount importance to establish a baseline for epidemiological studies, to determine changes in frequency, and to assess interventions. In developing countries, conventional methods for diagnosing dermatophytosis involve clinical examination, direct microscopic assessment of specimens, and culture.^15^ However, for more sensitive and time-saving diagnostics, DNA-based approaches such as real-time polymerase chain reaction (PCR) for the detection of fungi have been developed.^16^

 The prevalence of dermatophytosis was found to differ between genders. In our observation, men were generally found to be more vulnerable to dermatomycosis than women. The possible reason behind this observation is that men are more likely to engage in prolonged outdoor activities.^8^ In particular, we found that tinea cruris and tinea pedis prefer males over females. Tinea cruris is often associated with tinea pedis, as clothing gets contaminated when passing over the feet before it comes into contact with the groin. The predisposing factors for these conditions are obesity, excessive sweating, and a warm, moist environment.^15^ Taken together, these findings are consistent with the results of the majority of studies reported from different countries such as Brazil,^17^ Tunisia,^18^ India,^19^ Lebanon,^20^ and Iran.^8,21-24^

 The age of the studied group seemed to be a principal factor influencing the prevalence of dermatophytosis.^25^ Patients aged 25–44 years represent a significant proportion of cases in the current investigation, which can be explained by the fact that most adults at this age are employed. This result is somewhat consistent with the findings of other previous studies conducted in Iran.^8,21,24^ Among this age group, prolonged and vigorous outdoor activities play a significant role in exposing individuals to fungal pathogens.

 The main clinical entities among our studied patients were tinea corporis (27.2%), tinea cruris (26.6%), and tinea pedis (18.2%). Tinea corporis is currently the most pervasive clinical form of dermatophytosis in the Middle East.^15^ This form spreads mainly through human-to-human contact worldwide. Prior studies from Iran^8,23,26^ and India^19^ reported that tinea corporis was the most common form of dermatophytosis. Although *Microsporum* spp. were reported as the predominant agents of tinea corporis in Europe and many other parts of the world,^27,28^ in the present work, however, *T. tonsurans* was the main etiological agent of tinea corporis, followed by *T. rubrum*. Two investigations from Poland^29^ and India^30^ cited *T. rubrum* as the principal pathogen of tinea corporis. Similar to our results, a previous study from Tehran reported that *T. tonsurans* accounts for a significant proportion of tinea corporis cases.^8^ In northeastern Iran,^23^ tinea corporis was primarily caused by *T. mentagrophytes*, whereas *T. verrucosum* was frequently observed in Qazvin.^31^

 According to some previous surveys conducted in Iran, tinea cruris (groin) or jock itch was the second most common clinical manifestation of dermatophytosis.^8,21,22^ Patients with tinea cruris may have tinea pedis as well, and it has been speculated that tinea cruris is spread by hand from the tinea pedis infection.^32^ Tinea cruris is caused primarily by *T. rubrum* worldwide.^32^ However, *E. floccosum* still remains as the main causative agent of tinea cruris in various regions of Iran.^33-35^ Somewhat in contrast with the above-mentioned studies, *T. mentagrophytes* and *T. rubrum* were the major dermatophytes causing tinea cruris in the present work.

 As regards tinea pedis, it was ranked the third in prevalence, accounting for 18.2% of all types of ringworm in the current study. Previous investigations found higher frequencies of tinea pedis in Iran compared with our results.^8,22,33,36^ *T. rubrum* is the most prominent species implicated in tinea pedis worldwide.^15,28,37^ On the contrary, we observed *T. mentagrophytes* as the most frequent species among patients with tinea pedis, which is concordant with some reports from different regions of Iran.^8,21,22,33^ Animal contacts are considered to be the source of human infections since they are natural carriers of the zoophilic dermatophytes. Moreover, certain contributing factors including wearing socks and stockings may affect the development of tinea pedis.^38^

 Tinea unguium constituted 5.9% of all infections, most commonly pertaining to the age group 45–64 years old. The low frequency of onychomycosis is attributed to a shift from dermatophyte to molds and yeasts.^39,40^ In general, nail infection predominantly affects males and older adults, which is also observed in the current survey.^41^ Like our study, the most commonly encountered pathogen causing onychomycosis is *T. rubrum* throughout the world.^29,41-43^ A similar national trend has also been observed in Iran.^8,22,36^

 Tinea manuum is a superficial dermatophytic infection that involves one or both hands, sometimes including the palms. In addition to the hands, the feet are often involved. In the present work, it constituted 13.2% of all infections, which is higher than those reported by other Iranian studies.^8,22,33^ Contrary to the global^44^ and national trends,^21,22,33,36^ in which *T. rubrum* is the usual cause of tinea manuum, *T. tonsurans* was the most common species in the current study. Only one study in our country found *T. tonsurans* as the main causative agent of tinea manuum.^8^

 Regarding tinea capitis, it accounted for 3.65% of all infections, which is lower than those reported by other Iranian studies.^8,24,33,36^ Tinea capitis was for a long time the foremost dermatophytic infection in some parts of Iran.^40,45^ According to recent reports, tinea capitis incidences appear to be on the decline nationwide.^8,33,36^ In the current study, *T. tonsurans* was the most common causative agent of tinea capitis, which is in line with the findings of a previous work from Tehran.^35^ In contrast to these results, *T. verrucosum* was ranked as the most prevalent cause of tinea capitis in Isfahan.^40^ Contrariwise, *T. violaceum* and *T. schoenleinii* were frequently associated with tinea capitis in Mashhad.^23^ From an etiological standpoint, *T. tonsurans* dominates the disease in the United Kingdom, Canada, and Mexico, *M. canis* prevails in Australia and several parts of Europe, and *T. violaceum* is commonly isolated in North Africa, China, and South Asia.^27,46,47^

 We showed that tinea faciei manifested significantly more in the age group of 15–24 and due to *T. tonsurans*. Prior studies have given little or no attention to tinea faciei. The dominant species responsible for tinea faciei differ depending upon the geographic location and the potential animal reservoir.^48^ Unlike our findings, many studies from various cities of Iran found *T. mentagrophytes *as the most frequent causative agent.^21,22,36^ Similar to our results, *T. tonsurans* was the prevailing species in a previous study from Tehran.^33^ Infections caused by zoophilic species such as *M. canis* and *T. mentagrophytes *tend to occur more frequently in children because of their contact with pets including dogs, cats and rabbits.^48^ Pets are apparently less popular in Iran, and potential infection sources are either domestic animals or people.^33^ In the current work, beard and mustache infections were the least common infection type (0.4%), and the principal cause was *T. mentagrophytes*. Over the past years, Iran has witnessed a decrease in cases of tinea barbae. This was expected because nowadays men pay more attention to their personal hygiene.^21,22,33^ Similarly, tinea barbae is infrequent throughout the world.^15,48^

 The distribution of dermatophyte species varies considerably across different geographical regions. As mentioned earlier, the epidemiology of dermatophytes is influenced by various factors including, but is not limited to, climatic factors, occupation, migration, socioeconomic and lifestyle conditions, and the introduction of new therapeutic methods.^37^

 One major limitation of the current study was the retrospective nature of the analysis together with small sample sizes. Moreover, because only two hospitals were selected in this study, our findings may not be extrapolated to a whole region, or be used to make broad conclusions. Larger samples sizes together with inclusion of diverse locations would yield more accurate results in future investigations. Comprehensive information with regard to the occupational status and underlying comorbidities such as diabetes, cancer, and immunodeficiency would also be helpful since these conditions may lead to an increased risk of fungal infections.^49^

 In conclusion, understanding the epidemiology of dermatophytosis is critical for their prevention and therapy. In the present study, *T. mentagrophytes* had the highest prevalence compared to the rest of the dermatophytes. Furthermore, tinea corporis and tinea cruris were the most abundant clinical forms among the patients. We also observed that patients aged 25–44 years represented the greatest proportion of cases. Further studies with larger sample sizes and inclusion of diverse locations within the region would provide more reliable results. Finally, the use of molecular-based techniques such as PCR will not only reduce the turn-around time from that observed with traditional culture-based identification methods, but will also unveil the full spectra of dermatophyte species.
